# A cross-sectional study of the association between dynapenia and higher-level functional capacity in daily living in community-dwelling older adults in Japan

**DOI:** 10.1186/s12877-016-0400-5

**Published:** 2017-01-03

**Authors:** Masaki Iwamura, Masao Kanauchi

**Affiliations:** 1 Graduate School of Health Science, Kio University, 4-2-2 Umaminaka, Koryocho, Kitakatsuragigun, Nara 635-0832 Japan; 2 Department of Physical Therapy, Faculty of Health Science, Aino University, 4-5-4 Higashioda, Ibaraki, Osaka 567-0012 Japan

**Keywords:** Dynapenia, Sarcopenia, Frailty, TMIG-IC, Community-dwelling older adults

## Abstract

**Background:**

There are many reports that dynapenia, sarcopenia and frailty each have associations with bodily function or with Instrumental Activities of Daily Living (IADL). However, studies that compare all three conditions and their effects on IADL are lacking. The purpose of this study is to examine associations of sarcopenia, frailty, and dynapenia with IADL.

**Methods:**

Participants included 123 community-dwelling older adults (31 men, 92 women,) aged 65 years or older (75.0 ± 5.3 years) who were independent in IADL. In terms of physical function, measurements were performed for muscle mass, grip strength, walking speed, isometric knee extension strength, and unipedal standing. A questionnaire survey was carried out, the Tokyo Metropolitan Institute of Gerontology Index of Competence (TMIG-IC) was administered, and participants were asked about sense of fatigue and amount of activity.

**Results:**

Dynapenia was associated with classifications of both frailty and sarcopenia. In addition, sarcopenia had a sensitivity and specificity for dynapenia of 33 and 89%, respectively. Frailty had a sensitivity and specificity for dynapenia of 17 and 98%, respectively. Dynapenia was a significant independent related factor for the TMIG-IC (β = −0.21, *P* < 0.05).

**Conclusions:**

Dynapenia, more than sarcopenia or frailty, was related to difficulties with IADL; therefore, assessment of dynapenia should be given greater emphasis in evaluating the physical functioning of older adults.

## Background

As recent years have attested, Japanese societies are increasingly aging. Controlling the continually expanding social expense of this phenomenon is an urgent problem, as is reducing the proportion of older people needing care. It is therefore is necessary to lengthen healthy life expectancy. With respect to degenerative changes, the concept of frailty has attracted much attention recently.

Frailty is the foundation of the variety of functional declines associated with aging (lowering of reserve force) that are associated with increasing vulnerability to a variety of health problems. It has been said that frailty is an intermediate stage between an elderly person’s functioning normally and needing care [1].

Sarcopenia is a central component of frailty [2]; therefore, intervention in and evaluation of this condition may help prevent the progression to frailty. The term sarcopenia was coined by Rosenberg [3]. At first it was understood as “muscle atrophy with aging,” but its clinical operational criteria had not been established. For this reason, in 2010 the European Working Group on Sarcopenia in Older People (EWGSOP) recommended using the presence of both low muscle function (low physical performance or low muscle strength) and low muscle mass to diagnose sarcopenia [4]. In addition, in 2013, the Asia Working Group for Sarcopenia (AWGS) developed criteria in Asia [5], and their clinical application has since become possible in Japan. However, in recent years, reported changes associated with aging include decline in muscle strength occurring markedly more than decline in skeletal muscle mass [6]. Furthermore, it has been reported that physical function and health indicators were associated with walking speed and lower extremity muscle strength, but not with skeletal muscle mass [7, 8]. In addition, there are reports that a decrease in quantity of muscle contributes only 5–10% to diminishment of muscle strength. Therefore, evaluation of muscular strength, rather than muscle mass, is important [9, 10].

In 2008, the concept of dynapenia that was proposed by Clark et al. [11] and indicated a decline in muscle strength with aging highlighted the significance of this condition for assessing muscle strength [12, 13]. In 2012 an algorithm for dynapenia was presented by Manini et al. [14], and reports comparing sarcopenia with dynapenia begin to appear. The latter condition has been linked more strongly than the former to the ability to execute movements and to falling [15], as well as to mortality [16].

However, few reports have addressed the association between dynapenia and Instrumental Activities of Daily Living (IADL), and to date, no published studies have examined the associations of all three conditions (sarcopenia, dynapenia and frailty) with IADL. The present study aims to address these gaps.

This study’s hypothesis is that dyanapenia, which has shown a strong association with body function, also has a stronger association with IADL than does sarcopenia or frailty.

In addition to using the Unipedal Standing Test (UST), which is a useful test for predicting falls [17], we examined the definitions of sarcopenia, frailty and dynapenia as these relate to the Tokyo Metropolitan Institute of Gerontology Index of Competence (TMIG-IC), to evaluate the activity ability that is required to engage in independent living in the community [18].

## Methods

### Participants

Recruitment of participants was performed through resident associations, older adult meetings and preventive care establishments in Nishinomiya, Hyogo Prefecture and Ikeda, Osaka Prefecture and the town of Ikaruga, in Nara. The participants included 123 older adults (31 men, 92 women) aged 65 years or older (mean 75.0 ± 5.3 years) who had not received a Certification of Needed Support and Long-Term Care for older adults.

Exclusion criteria were: use of a body implant apparatus, including a pacemaker or artificial joint; any condition that presented a clear physical disability (bone fracture, amputation, difficulty walking); severe dementia (that would create difficulty with understanding instructions) or a severe heart condition not controlled by medication.

The study was approved (H26-11) by the Kio university research ethics committee.

### Measurements

#### Physical function

We performed measurements of physical function, muscle mass, grip strength, walking speed, isometric knee extension strength, and UST. Muscle mass measurement was carried out with the In Body 430 (In Body Japan, Tokyo, Japan), using the Bioelectrical Impedance Analysis (BIA) method. Measurement of posture involved standing barefoot on the measuring equipment and gripping the measurement terminals with both upper limbs. Grip strength measurements were made twice on each side with a dynamometer (Digital Handgrip Meter KEEP, MACROSS Inc. Tokyo, Japan). The average of the left and right maximum values were taken. Walking speed was measured for a distance of 6 m, the acceleration path and deceleration path were set as 1 m, and measured in tenths of a second, and participants walked the path twice, and the mean speed was taken. Isometric knee extension strength measurements were performed using a belt-fixed handheld dynamometer (μ-tas F1, Anima Co, Tokyo, Japan).

For measuring isometric knee extension strength, the participants sat on a training bench and adjusted the position of their gluteal regions so that the leg of the bench was posterior to the lower limb being measured. The height of the training bench was set so that each participant’s legs hung from the bench (with feet not touching the floor). The participants maintained their trunk in a perpendicular position with both hands touching the bench surface on either side of the trunk, and knee joint set at an angle of 90°. The belt anchoring site was set to 4 on the horizontal fingers, then the medial malleolus and the lateral malleolus measurements were carried out twice on each side, maintaining 3 s at maximum output at that position. The average of the left and right maximum values was retained for each participant. For measuring the UST, the subjects were instructed to keep their legs from touching and to maintain a unipedal stance for as long as possible. This was measured for 60 s as the upper limit. Two measurements were taken on each side and the average of the left and right maximum values were taken.

#### Questionnaire

The questionnaire included the TMIG-IC and questions about fatigue and amount of activity that were partially modified by Shinkai et al. [19] based on the Fried et al. [20] questionnaires. The TMIG-IC was developed as a comprehensive evaluation of the activity capacity of healthy older adults living in the region. It is a multidimensional 13-item scale that consists of three subscales: IADL (five items), intellectual activity (four items) and social role (four items); there is sufficient verification of this questionnaire’s reliability and validity [18].

#### Operational definition of each decision

To derive the cut-off values used for the sarcopenia definition with reference to the presented values of AWGS [5], the muscle mass value was obtained by dividing the sum of the limbs’ muscle mass by the square of height of the skeletal muscle mass index (SMI): 7.0 kg/m^2^ for men and 5.4 kg/m^2^ for women. The cutoff for grip strength in men was 26 kg and in women it was 18 kg, and a 6 m walking speed of 0.8 m/sec was set as the cutoff for both men and women. We defined sarcopenia as the presence of both low muscle function (low physical performance or low muscle strength) and low muscle mass.

We used the components identified by Freid et al. [20] for frailty: (1) shrinking: measured as weight loss; (2) weakness: measured as grip strength; (3) poor endurance and energy: measured by questionnaires; (4) slowness: measured as decreased walking speed and (5) low physical activity level: measured by questionnaires, defined as endorsing three or more items. Incidentally, the cut-off values for grip strength and walking speed were the same as those for sarcopenia.

To define dynapenia we used cut-off values that included isometric knee extension strength of 18.0 kg in men 16.0 kg in women. The values were calculated as described by Assantachai et al. [21], who monitored 2149 men and women over 60 years of age for a period of 2 years.

The cut-off value for UST was set to 30 s; this has been reported by Hurvitz et al [17] to be useful for fall prediction. We defined “static balance disorder” as UST less than 30 s.

### Statistical analysis

We compared the results of measurements between men and women using the Student’s *t*-test. The relevance of dynapenia and each of the other conditions (sarcopenia, frailty) was analyzed using the chi-square test. We used Pearson’s correlation coefficient to examine the correlations between the isometric knee extension strength and each of the other factors (SMI, walking speed, grip strength, UST). We used simple linear regression analysis to extract the factors associated with the TMIG-IC. It should be noted that we assigned the TMIG-IC outcome as the dependent variable, and the other factors (sarcopenia, frailty, dynapenia, age, and gender) as the independent variables. We used multiple regression analysis to determine which independent variables were relevant to the TMIG-IC scores. All statistical analyses were performed using SPSS version 20 for Windows (IBM Japan, Tokyo). A *P* value of less than .05 (2-tailed) was considered statistically significant.

## Results

Participant characteristics and other measurement data are presented in Table 1.

**Table 1:** Participant characteristics and other measurement data

	Total (*N* = 123)	Men (*N* = 31)	Women (*N* = 92)	*P*-value
Physical Characteristics				
Age	75.4 ± 5.2	78.2 ± 6.2	74.5 ± 4.5	0.00
Height (m)	1.5 ± 0.1	1.6 ± 0.1	1.5 ± 0.1	0.00
Weight (kg)	53.3 ± 8.1	57.3 ± 8.0	51.9 ± 7.7	0.00
Body fat (%)	29.6 ± 7.5	23.6 ± 6.9	31.6 ± 6.6	0.00
Disease				
Musculoskeletal disease (N)	18	1	17	
Cardiovascular disease (N)	34	8	26	
Endocrine disease and metabolic disease (N)	18	2	16	
Digestive disease (N)	11	6	5	
Neoplasm (N)	9	4	5	
Skeletal Muscle Mass				
Upper limbs (kg)	3.4 ± 0.8	4.3 ± 0.7	3.1 ± 0.5	0.00
Lower limbs (kg)	11.1 ± 2.3	13.6 ± 2.1	10.4 ± 1.6	0.00
Total (kg)	29.4 ± 8.4	35.6 ± 8.6	27.3 ± 7.2	0.00
SMI (kg/m^2^)	6.2 ± 0.8	6.9 ± 0.6	5.9 ± 0.7	0.00
Physical Function				
Knee extension muscle strength (kgf)	21.5 ± 7.7	28.8 ± 9.3	19.1 ± 5.1	0.00
Handgrip strength (kg)	22.3 ± 5.4	28.7 ± 5.3	20.1 ± 3.3	0.00
Walking speed (m/sec)	1.4 ± 0.3	1.3 ± 0.3	1.4 ± 0.4	0.04
Unipedal standing (sec)	32.8 ± 21.6	31.0 ± 19.6	33.5 ± 22.3	0.58
Judgement result				
Sarcopenia, n (%)	20(16)	8(26)	12(13)	0.09
Dynapenia, n (%)	30(24)	4(13)	26(28)	0.08
Frailty, n (%)	7(6)	2(6)	5(5)	0.81
Static balance ability decline, n (%)	61(50)	16(52)	45(49)	0.57
TMIG-IC (Score)	11.8 ± 1.5	11.4 ± 1.7	11.9 ± 1.5	0.10

*SMI* skeletal muscle mass index, *TMIG-IC* the Tokyo Metropolitan Institute of Gerontology Index of Competence

All of the values for the physical features and a variety of physical function measurement results were significantly higher in men than in women, except for UST. The dynapenia classification was associated with both of the other classifications (sarcopenia, *P* < 0.01; frailty, *P* < 0.01) (Table 2).

**Table 2:** Interrelation of sarcopenia and frailty with dynapenia

	Dynapenia (*N* = 30)	Non-Dynapenia (*N* = 93)	Sig (*χ*2)
Sarcopenia (*N* = 20)	10	10	0.006
Non-Sarcopenia (*N* = 103)	20	83	
Frailty (*N* = 7)	5	2	0.009
Non-Frailty (*N* = 116)	25	91	

All tests were analyzed using chi-square test

Correlations of knee extension muscle strength and the other physical functions, and sensitivity and specificity for dynapenia of frailty and sarcopenia are shown in Table 3 and Table 4.

**Table 3:** Correlation of knee extension muscle strength with SMI, walking speed and handgrip strength

	Knee extension muscle strength	
	Men (*N* = 31)	Women (*N* = 92)
SMI	0.41 (*P* = 0.02)	0.10 (*P* = 0.33)
Walking speed	0.35 (*P* = 0.051)	0.17 (*P* = 0.092)
Handgrip strength	0.53 (*P* = 0.002)	0.36 (*P* < 0.001)
Unipedal standing test	0.61 (*P* < 0.001)	0.37 (*P* < 0.001)

*SMI* skeletal muscle mass index; Unipedal standing test shows unipedal standing retention time

**Table 4:** Sensitivity and specificity for dynapenia

	Sensitivity	Specificity
Sarcopenia	33%	89%
Frailty	17%	98%

Each definition includes participants who have both sarcopenia and frailty

Results of the single regression analysis testing the associations between the TMIG-IC and the other factors (sarcopenia, frailty, dynapenia, static balance disorder, age and sex) were computed. Dynapenia, static balance disorder and age were significantly related factors. We performed multiple regression analysis assigning the significantly relevant variables (dynapenia, static balance disorder and age) as independent variables and TMIG-IC score as the dependent variable. Results of the analysis extracted dynapenia as the most significant independent variable (β = −0.21, *P* < 0.05) (Table 5).

**Table 5:** Association between TMIG-IC score and significant factors from multivariable linear regression

Variable	B	β	*P*-value	95%CI of B value	
Dynapenia	-0.75	-0.21	0.015	-1.35	-0.15
Age	-0.50	-0.17	0.066	-0.10	0.003
Static balance disorder	-0.56	-0.19	0.049	-1.12	-0.001

Independent variables are variables that showed significant difference in single linear regression analysis

## Discussion

In this study, 123 community-dwelling older people were evaluated for sarcopenia, dynapenia, and frailty, and their status with regard to these three conditions was examined for associations with TMIG-IC scores. Only dynapenia was extracted as a meaningful factor. In preceding studies, there have been reports that sarcopenia and frailty were associated with IADL and mortality [22, 23], but no studies had examined potential interrelationships of frailty, dynapenia and sarcopenia within the same investigation.

Reports that compare sarcopenia with dynapenia are beginning to appear. For example, one study tracked these conditions’ association with falls in 674 community-dwelling older individuals [24], and another examined associations with cognitive functional disorders for older community-dwellers in Taiwan [25]. Yet another study investigated risk factors for mortality in 1149 older people in Brazil [16]. Dynapenia was extracted as a stronger predictor than sarcopenia in all three studies. In addition, according to Kim et al. [26], muscle strength of limbs is linked more strongly to physical performance than is muscle mass.

In contrast, relationships between TMIG-IC scores and sarcopenia and frailty have been found in previous studies [22, 23], but there were no such relationships observed in this study. Previously, the TMIG-IC has been analyzed using a cut-off value of 10 points or less to distinguish “independence/non-independence” but this study did not confirm such classification. In other words, there is possibility that the low scores (10 points or less) on the TMIG-IC were more related to sarcopenia and frailty, but the participants of the “activity diminished ability preliminary group” who had a slight drop of 11 to 12 points did not have a significant link to sarcopenia and frailty. This may be due to the fact that the average TMIG-IC score in our participants was 11.8 points, which is somewhat high.

The associations between dynapenia and each of the other two conditions (sarcopenia and frailty) were relevant. The reason for this may be that isometric knee extension strength was used in the dynapenia definition, and grip strength was used in both the sarcopenia and frailty definitions; therefore, the observed link may be a result of a moderate correlation between the two strength measures. But the sensitivity of the sarcopenia and frailty definitions for the dynapenia definition was 33 and 17%, respectively. In this study, the 30 participants had dynapenia, but 20 of these 30 did not have sarcopenia, and 25 of the 30 did not exhibit frailty. It is necessary to perform an assessment of dynapenia separately from that of sarcopenia and frailty, and in this regard knee extension strength is more important than handgrip strength.

Handgrip measurement has the advantage of being simple and easy, and is sometimes used as an index of muscular strength for the whole body, but this measure is only weakly correlated with lower limb muscular strength [27].

The correlation of handgrip strength with isometric knee extension strength of the women in this study was weak to moderate (0.36); therefore, one cannot deny the possibility that handgrip measurement is insufficient for assessing fall risk.

In contrast, there are many reports that lower limb strength, particularly knee extension strength, is strongly related to locomotion, balance ability and IADL [28–30]. In recent years, the use of belt fixation-type handheld dynamometers such as employed in this study has spread. Because these devices improve measurement accuracy, the accumulation of reliable data is now possible. Therefore, we measured dynapenia using knee extension strength and were able to relate this measurement to the life functions of older people.

For assessing dynapenia, Manini et al. [14] suggested that the diagnostic algorithm should begin by screening participants who are over 60 years of age, and that those who have sufficiently severe risk factors for the development of dynapenia should be referred for a knee extension strength assessment. In this study, all participants performed the isometric knee extensor strength test. The reason for this was that there was a risk of dynapenia in all participants, whose average age was 75.4 ± 5.2 years. Also, I used a cut off value exhibited in Asia [21] thought to be a frame and the muscular strength similar to Japanese. But the appropriate cut-off value is not yet clear, and whether it is necessary to compensate by weight or height are necessary verifications.

In addition, there have been recent reports that dynapenic obesity, (co-occurring obesity and dynapenia) has a similar relationship to that seen with ADL in the elderly [31]. Therefore it is necessary in the future to clarify a cut-off level for diagnosing dynapenia, and to examine what kind of influence dynapenia and dynapenic obesity have on a life functions and mortality of older people.

A limitation of this study is that the results cannot be applied to all of the older adults in this community, because the participants were elderly persons who applied to a health program in a certain area and are therefore not representative of the general community. In addition, this study used provisional criteria for dynapenia; the criterion validity remains to be verified in the future. In addition, this study’s results may have been affected by participants’ meal and fluid intake on the day before the measurements. Because this study used the BIA method of estimating muscle quantity from fat-free mass, rather than more direct measures of muscle substance, such as computed tomography or magnetic resonance imaging, errors in muscle measurement may have been introduced.

## Conclusion

This study examined associations of sarcopenia, frailty and dynapenia with IADL. Dynapenia was associated with sarcopenia and frailty, but sarcopenia and frailty had a low sensitivity to dynapenia. In addition, dynapenia had a stronger association with IADL of the community-dwelling older participants than did sarcopenia or frailty.

## Abbreviations

AWGS: Asia Working Group for Sarcopenia
BIA: Bioelectrical Impedance Analysis
EWGSOP: European Working Group on Sarcopenia in Older People
IADL: Instrumental Activities of Daily Living
SMI: Skeletal muscle mass index
the TMIG-IC: The Tokyo Metropolitan Institute of Gerontology Index of Competence
UST: Unipedal Standing Test
